# The Inner Centromere Protein (INCENP) Coil Is a Single α-Helix (SAH) Domain That Binds Directly to Microtubules and Is Important for Chromosome Passenger Complex (CPC) Localization and Function in Mitosis*

**DOI:** 10.1074/jbc.M115.645317

**Published:** 2015-07-14

**Authors:** Kumiko Samejima, Melpomeni Platani, Marcin Wolny, Hiromi Ogawa, Giulia Vargiu, Peter J. Knight, Michelle Peckham, William C. Earnshaw

**Affiliations:** From ‡ The Wellcome Trust Centre for Cell Biology, University of Edinburgh, King's Buildings, Max Born Crescent, Edinburgh EH9 3BF, Scotland, United Kingdom and; § The Astbury Centre for Structural Molecular Biology, University of Leeds, Leeds LS2 9JT, United Kingdom

**Keywords:** centromere, microtubule, mitosis, mutant, protein structure, CPC, INCENP, chromosome passenger complex, coiled coil, single α-helix

## Abstract

The chromosome passenger complex (CPC) is a master regulator of mitosis. Inner centromere protein (INCENP) acts as a scaffold regulating CPC localization and activity. During early mitosis, the N-terminal region of INCENP forms a three-helix bundle with Survivin and Borealin, directing the CPC to the inner centromere where it plays essential roles in chromosome alignment and the spindle assembly checkpoint. The C-terminal IN box region of INCENP is responsible for binding and activating Aurora B kinase. The central region of INCENP has been proposed to comprise a coiled coil domain acting as a spacer between the N- and C-terminal domains that is involved in microtubule binding and regulation of the spindle checkpoint. Here we show that the central region (213 residues) of chicken INCENP is not a coiled coil but a ∼32-nm-long single α-helix (SAH) domain. The N-terminal half of this domain directly binds to microtubules *in vitro*. By analogy with previous studies of myosin 10, our data suggest that the INCENP SAH might stretch up to ∼80 nm under physiological forces. Thus, the INCENP SAH could act as a flexible “dog leash,” allowing Aurora B to phosphorylate dynamic substrates localized in the outer kinetochore while at the same time being stably anchored to the heterochromatin of the inner centromere. Furthermore, by achieving this flexibility via an SAH domain, the CPC avoids a need for dimerization (required for coiled coil formation), which would greatly complicate regulation of the proximity-induced trans-phosphorylation that is critical for Aurora B activation.

## Teaser

**Background:** INCENP is predicted to have a coiled coil domain.

**Results:** The coil is actually a stable single α-helix (SAH) domain that is highly extendable and directly binds microtubules.

**Conclusion:** This flexible *dog leash* may allow Aurora B to associate with dynamic targets in the outer kinetochore.

**Significance:** The SAH domain allows CPC flexibility without requiring complex dimerization.

## Introduction

INCENP^6^ is the scaffolding protein upon which the chromosomal passenger complex (CPC) assembles (1–3). The N-terminal region of INCENP assembles a three-helix bundle with Survivin and Borealin (4) that contributes to targeting the CPC to inner centromeres via haspin-mediated phosphorylation of histone H3 (5–7). This region of INCENP also contributes to CPC localization by binding to HP1 and to microtubules. The IN box, a conserved motif near the C terminus of INCENP, is responsible for binding and activating Aurora B kinase (3, 8). The central portion of INCENP is predicted to form a coiled coil spacer between the localization and activation modules (3) and has been proposed to act as a “dog leash,” allowing Aurora B tethered to chromatin to phosphorylate substrates within a constrained region (9).

This tethering of Aurora B is critical for the regulation of chromosome alignment and the correction of kinetochore attachment errors. Spindle tension causes the elastic chromatin of the inner centromere to stretch, allowing sister kinetochores to move away from the inner centromere where INCENP is concentrated during prometaphase and metaphase. As first proposed for budding yeast and later confirmed in mammalian cells, this centromere stretch moves kinetochore targets of Aurora B away from regions of high kinase concentration and decreases their phosphorylation, thereby stabilizing kinetochore-microtubule interactions (10–12). In misattached chromosomes, which do not exhibit comparable centromere stretch, Aurora B remains in closer proximity to the outer kinetochore. This allows the kinase to phosphorylate key kinetochore components, causing them to release microtubules (13–15).

This correction mechanism is now well accepted, but what is less clear is how exactly INCENP achieves the dynamic flexibility to allow Aurora B to extend into the outer kinetochore and to track with kinetochore components at the dynamic microtubule interface. For example, because the Ndc80 and Ska complexes are composed of relatively rigid helical bundles (16, 17), it is not clear how Aurora B is able to associate with them if they undergo conformational changes on the dynamically growing and shrinking kinetochore-associated microtubules.

Here we show that the central region of INCENP is not a coiled coil but instead is a single α-helix (SAH) domain similar to that found in myosin 10 and many other proteins (18–21). The N-terminal portion of this SAH is capable of binding directly to microtubules. Furthermore, SAH domains are highly extensible, and by analogy with the myosin SAH domain (20), it is likely that extension of the relatively lengthy INCENP SAH might allow the IN box with its bound Aurora B to undergo excursions of up to ∼80 nm under relatively light loads. These data support the suggestion that the INCENP coil functions as a dog leash that allows Aurora B to “wander” across a substantial target area to reach its substrates (9). By using an SAH rather than a coiled coil to achieve this flexibility, INCENP avoids the requirement for dimerization, which would significantly complicate the currently accepted mechanism of proximity/clustering-induced activation of the CPC (12, 22–24).

## Experimental Procedures

### 

#### 

##### Protein Expression and Purification

All proteins were expressed in *Escherichia coli* BL21 Rosetta 2 (Novagen) and purified using a nickel-nitrilotriacetic acid affinity chromatography column. Proteins were dialyzed against 150 mm NaCl, 20 mm Tris, 1 mm DTT, pH 8.0 and proteolyzed for 2 h at room temperature using ULP1 recombinant small ubiquitin-like modifier protease in a substrate to enzyme ratio of 100:1. Next, proteins were purified on ion-exchange columns using an ÄKTA system. The purest fractions were combined and concentrated, resulting in a 1–2 mg/ml protein solution. Purified protein was dialyzed against 100 mm NaCl, 10 mm sodium phosphate, pH 7.4 and snap frozen in liquid nitrogen for long term storage at −80 °C.

##### Mass Spectrometry

Protein samples (∼0.2 ml; 20 μm) were dialyzed (G-Biosciences dialyzers 2-kDa-molecular mass cutoff) overnight against 50 mm ammonium acetate, pH 7.4 and analyzed by TOF MS analysis (The University of Leeds Mass Spectrometry Facility).

##### Circular Dichroism (CD) Spectroscopy

CD measurements were performed on an Applied Photo Physics Chirascan CD spectropolarimeter with a 0.1-cm-path length quartz cuvette in 0.1 m NaCl, 10 mm sodium phosphate, pH 7.4 buffer. Data were collected every 1 nm with 30-s averaging time, each measurement being an average of two repeated scans. Data presented are averaged from at least two separate measurements of different protein preparations. Thermal measurements were performed in a temperature range from 10 to 85 °C with a 0.7 °C/min heating rate with data acquisition every 1 °C and 20-s averaging time. The sample cooling rate prior to measurement of refolded protein was ∼ 2 °C/min. The mean residue molar ellipticity of proteins was calculated as described (25). The helical content of proteins was calculated from values of the amide *n*π* transition at 222 nm ([MRE_222_]) as described previously (25). Protein concentration was measured by absorption at 280 nm. Absorption coefficients were obtained from ProtParam software. Standard concentrations were in the range of 10–20 μm. In the salt dependence experiments, stock buffer (5 m NaCl, 10 mm sodium phosphate, pH 7.4) was mixed with stock protein solution to obtain desired salt and protein (10 μm) concentration.

##### cDNA Constructs

Sequences encoding putative full-length INCENP SAH domain (UniProt ID P53352; *Gallus gallus*; residues 503–715) and its N-terminal (residues 503–597) and C-terminal (residues 598–715) fragments were subcloned into the pET28a SUMO vector (26) to introduce an N-terminal His tag and small ubiquitin-like modifier protein for increased expression and solubility. For all constructs, a tryptophan residue was added to the C terminus to enable *A*_280_ concentration measurements.

INCENP SAH mutant constructs were based on triple affinity purification (TrAP)-tagged INCENP^WT class I^ under control of an SV40 promoter that is insensitive to doxycycline repression (27). The TrAP tag incorporates His, streptavidin-binding peptide, and S tags and can be monitored by immunoblotting and immunofluorescence using a monoclonal antibody recognizing the streptavidin-binding peptide tag (28, 29). GFP was inserted in front of the TrAP tag to visualize the mutants. Silent mutations were introduced into INCENP cDNA to create BamHI, EcoRI, and HindIII sites around SAH domain so that the SAH region can be easily modified. Wild type SAH, half-SAH, double SAH, and double MyoM SAH (30) cassettes were synthesized at Geneart (Life technologies) and cloned into the GFP-TrAP-INCENP constructs.

##### Cell Culture

DT40 cells were grown in RPMI 1640 medium supplemented with 10% FBS, 1% chicken serum and maintained in 5% CO_2_ at 39 °C. Doxycycline at a final concentration of 500 ng/ml was added to the culture medium to repress transcription of the promoter-hijacked endogenous INCENP locus (29). HeLa Kyoto cells were grown in Dulbecco's modified Eagle's medium supplemented with 10% fetal calf serum, 0.2 mm l-glutamine, 100 units/ml penicillin, and 100 μg/ml streptomycin.

##### Immunoblotting

Whole cell lysates were prepared, and the equivalent of 0.5–1 × 10^6^ cells was loaded onto a polyacrylamide gel. SDS-PAGE and immunoblotting were performed following standard procedures. Donkey anti-mouse or -rabbit IRdye 800CW was used for analysis using a LI-COR Biosciences Odyssey quantitative fluorescence imager.

##### Indirect Immunofluorescence Microscopy

All fixation, permeabilization, and immunostaining were performed at room temperature as described previously (31). Cells attached on polylysine-coated coverslips were fixed in a 3.7% formaldehyde, PBS solution for 10 min and permeabilized in PBS, 0.15% Triton X-100 for 4 min. Cells were blocked in 10% normal donkey serum for 1 h at room temperature prior to antibody incubations. Antibodies used were α-tubulin antibody (B512 or DMIA, Sigma-Aldrich); anti-H3Ser^10^ph (Millipore); anti-GFP (Life Technologies); anti-HEC1 mouse monoclonal (Abcam); anti-DSN1ph (32); anti-H3Ser^28^ph (33); and rabbit polyclonal (WCE1186), anti-INCENP (3D3), anti-Aurora B, and anti-CENP-T, which were described previously (1, 27, 34). All affinity-purified donkey secondary antibodies (labeled with either FITC, Alexa Fluor 488, TRITC, Alexa Fluor 594, or Cy5) were purchased from Jackson ImmunoResearch Laboratories.

##### siRNA against Human INCENP

RNAi experiments were performed using annealed siRNA oligos (Qiagen) diluted in serum-free Opti-MEM and transfected using HiPerFect reagent (Qiagen) according to the manufacturer's protocol. HeLa cells were seeded on coverslips at a concentration of 1 × 10^5^ cells/ml, and diluted siRNA was added to cells so that the final concentration of siRNA was 40 nm. Plasmids encoding either GFP-TrAP-GgINCENP^WT SAH^, GFP-TrAP-GgINCENP^Double SAH^, or GFP-TrAP-GgINCENP^N-half SAH^ were transfected for 24 h prior to fixation. Coverslips were fixed at 30–34 h. INCENP siRNA oligo was 5′-AGATCAACCCAGATAACTA-3′ (35). For control transfections, non-silencing random scrambled siRNA oligos were used at the same concentration.

##### Image Analysis and Quantification

Quantifications of H3Ser^28^ph, H3Ser^10^ph, and DSN1ph were carried out as follows. Deconvolved images were imported into OMERO (36), and segmentation of centromere foci (anti-centromere antibodies, Cy5, reference channel) or chromatin (DAPI, 435 reference channel) performed using Otsu segmentation implemented in Matlab. Masks stored in OMERO were then used to calculate background-corrected intensities, which were output into a comma-separated value file for plotting in Excel.

##### Growth Curves

Growth curves were generated by seeding cells at a concentration of 2 × 10^5^ cells/ml at 39 °C (unless otherwise stated). Cell counting was performed every 24 h for a total of 96 h. To avoid overgrowth, cells were diluted to 2 × 10^5^ cells/ml every 24 h. The cell number at each time point was multiplied by the appropriate dilution factor to get a true count.

##### Microtubule Co-sedimentation Assay

Tubulin (Cytoskeleton Inc.) was used for the generation of polymerized microtubules according to the manufacturer's instructions. Taxol-stabilized microtubules (18 μm tubulin dimer) were incubated at room temperature for 10 min with 1 μm protein in a 50-μl reaction volume in BRB80 buffer (80 mm PIPES, pH 6.9, 1 mm EGTA, 1 mm MgCl_2_) with 100 mm NaCl and 4 mm DTT in the presence of 20 μm Taxol. The reaction was then layered onto a 250-μl glycerol cushion buffer (BRB80 buffer, 50% glycerol, 4 mm DTT) and ultracentrifuged for 10 min at 434,400 × g in a Beckman TLA 100.3 rotor at 25 °C. Pellets and supernatants were analyzed by SDS-PAGE. Gels were stained with Coomassie Blue, and protein quantification was performed with NIH ImageJ. Normalized binding data were obtained by dividing the values of the pellet fraction by the sum of pellet and supernatant.

## Results

### 

#### 

##### GgINCENP^503–715^ Has Properties Expected of a Stable SAH Domain and Is Not a Coiled Coil

**FIGURE 1.**
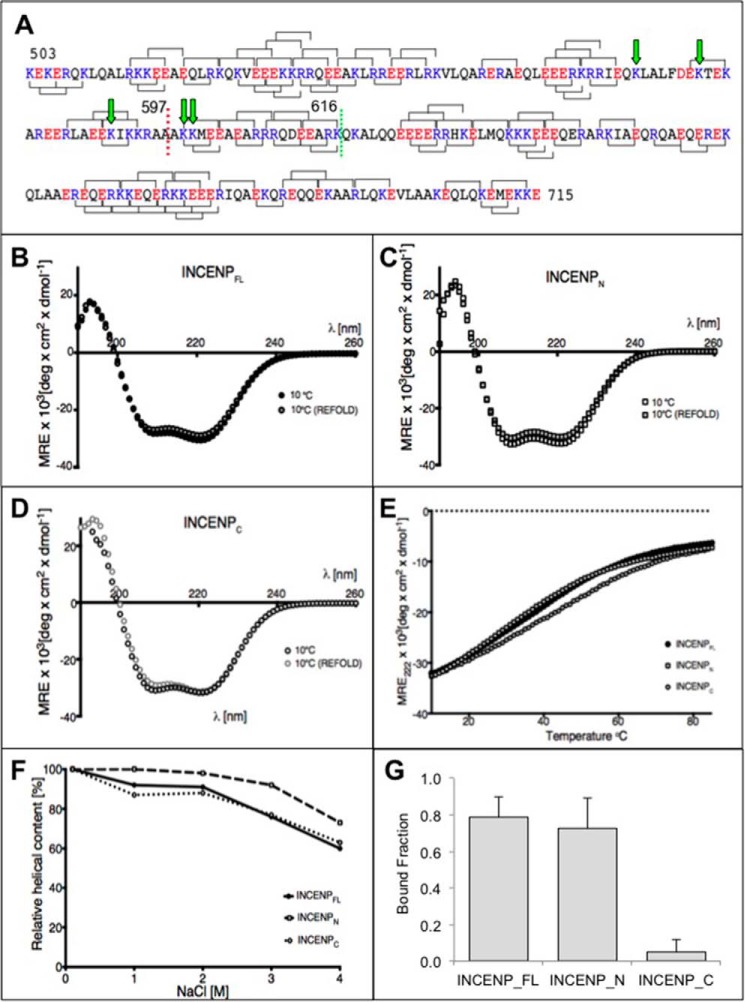
**Residues 503–715 from GgINCENP form an SAH domain in which the N-terminal half of SAH (residues 503–597) directly binds to microtubules *in vitro*.** *A*, the predicted SAH domain from INCENP. Acidic residues (Glu + Asp) are shown in *red*. Basic residues (Lys + Arg) are shown in *blue*. Potential *i*, *i* + 4 ionic interactions between Glu and Lys or Glu and Arg residues are shown as *brackets between* residues *above* the sequence, and potential *i*, *i* + 3 interactions are shown as *brackets below*. The positions of residues 597 and 616 are indicated *(red dotted line* and *green dotted line*, respectively). *Green arrows* point to corresponding residues reported to be modified in PhosphoSitePlus. The N-terminal SAH construct (*INCENP_N_*) consisted of residues 503–597, and the C-terminal SAH construct (*INCENP_C_*) consisted of residues 598–715. *B–D*, CD spectra for the full-length SAH construct from INCENP (*INCENP_FL_*) (*B*), N-terminal (*C*), and C-terminal (*D*) constructs. Two spectra are shown for each, the first at 10 °C prior to heating and the second at 10 °C after heating to 85 °C and allowing the peptide to refold. *E*, the thermal melt curves for all three constructs. The change in the mean residue ellipticity (*MRE*) value at 222 nm, which reports on the α-helical content of the protein, is plotted against temperature. *deg*, degrees. *F*, the response of all three constructs to increasing salt concentration. Helical content has been normalized to the value at 0.1 m NaCl (10 °C). *G*, average of bound fraction (*error bars* show +S.D.) of INCENP SAH proteins co-sedimenting with microtubules were obtained from four indepenent experiments.

The middle region of INCENP links the N-terminal centromere/microtubule-targeting domains with the C-terminal Aurora B regulatory domain. This middle region, GgINCENP^503–715^, has been predicted to form a coiled coil structure that is required for microtubule binding and microtubule-induced activation of Aurora B (24, 37). Subsequent detailed analysis of the INCENP sequence revealed numerous charged residues at positions of the heptad repeat that would disrupt coiled coil formation. We predicted that GgINCENP^503–715^ is not a coiled coil but might instead form a stable SAH domain (Fig. 1*A*) (19, 38). SAH domains are characterized by a highly helical secondary structure, non-cooperative thermal unfolding, and the ability of the peptide to completely refold after thermal denaturation. In addition, SAH domains remain helical up to fairly high salt concentrations (18).

To test this hypothesis, we prepared recombinant proteins containing the full-length SAH (INCENP^503–715^), the N-terminal SAH (INCENP^503–597^), and the C-terminal SAH (INCENP^598–715^). The exact position of the break in the sequence was based on analysis of potential ionic interactions between charged amino acid residues so that it did not disturb any of these potential bonds. We then performed CD measurements to investigate the secondary structure of these protein fragments over a range of temperature and salt conditions and looked at their ability to form monomers or dimers by measuring their molecular weight by mass spectrometry.

CD spectra revealed that all three proteins were highly helical at 10 °C at 0.1 m NaCl (Fig. 1, *B–D*). According to this analysis, INCENP^503–715^ was 85% helical, whereas INCENP^503–597^ and INCENP^598–715^ were ∼90 and 88% helical, respectively. All three constructs melted non-cooperatively as expected for SAH domains (Fig. 1*E*) and refolded after cooling to at least 90% of their initial helical content measured at 10 °C (Fig. 1, *B–D*). All three constructs remained highly helical up to 2 m NaCl, and then helical content decreased to ∼60% at 4 m NaCl (Fig. 1*F*), demonstrating the salt-resistant nature expected of an SAH domain (18). The helical nature of INCENP^503–597^ was slightly more resistant to increasing salt concentrations compared with INCENP^503–715^ and INCENP^598–715^. Mass spectrometry analysis confirmed that all of the studied constructs are monomeric with molecular masses of 12, 15.2, and 26.9 kDa for INCENP^503–597^, INCENP^598–715^, and INCENP^503–715^, respectively (data not shown).

All of the above strongly suggest that the middle region of INCENP is an SAH domain and not a coiled coil as proposed previously. Consequently, intact INCENP is likely a monomer and not a dimer, which has implications for its mechanisms of action.

##### The Full-length SAH and Its N-terminal Half Bind Directly to Microtubules in Vitro

The INCENP putative coil domain has been shown previously to be important for microtubule binding (24, 37, 39, 40). However, it remained unclear whether the SAH domain alone can bind to microtubules directly. We therefore used purified INCENP^503–715^, INCENP^503–597^, and INCENP^598–715^ recombinant proteins to perform microtubule co-sedimentation assays (41). INCENP^503–715^ and INCENP^503–597^ bound to microtubules (Fig. 1*G*), whereas the C-terminal portion of the SAH region, INCENP^598–715^, did not bind to microtubules in this assay. Thus, the INCENP SAH binds directly to microtubules, and the microtubule binding activity resides mainly in its N-terminal region.

##### Establishment of Various INCENP SAH Mutant Cell Lines

**FIGURE 2.**
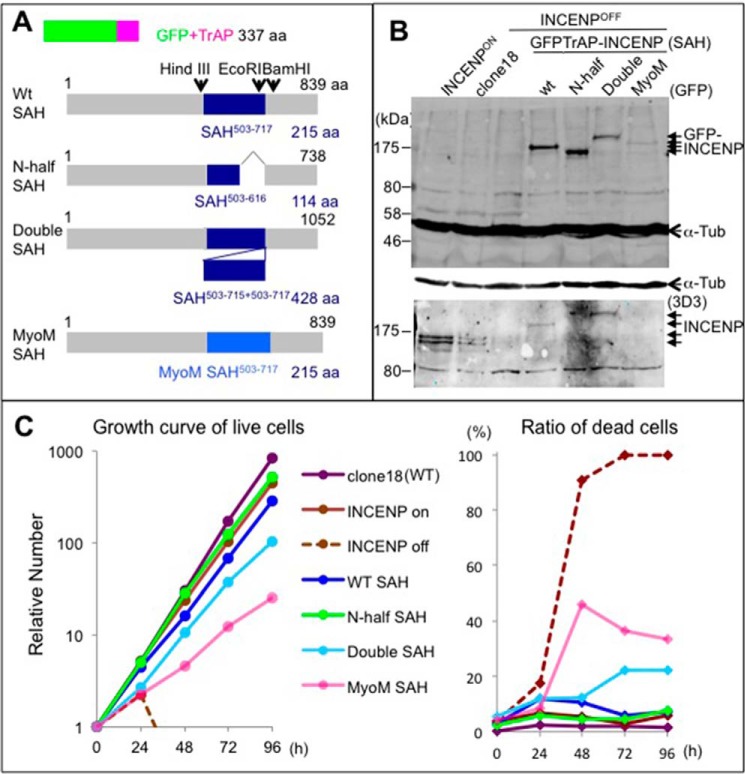
**DT40 cells stably expressing INCENP SAH mutants can proliferate in the absence of endogenous INCENP.** *A*, diagram of INCENP SAH domain swap mutants. Exchange of SAH domains was facilitated by the creation of HindIII, EcoRI, and BamHI sites that did not affect the amino acids sequence. A GFP-TrAP tag was attached to the N terminus of INCENP class I cDNA to visualize the proteins. *aa*, amino acids. *B*, expression of INCENP domain swap mutants in stable cell lines 26 h following addition of doxycycline in the medium. Apparently GgINCENP^617–717^ is highly antigenic. All our INCENP antibodies have epitopes in this region including the 3D3 antibody (monoclonal antibody against GgINCENP). Consequently, GFP-TrAP-INCENP with the N-terminal half of SAH or MyoM SAH is not detected by the 3D3 monoclonal antibody. The *lower panel* shows that GFP-TrAP-INCENP with the WT SAH was expressed at levels similar to those of the endogenous INCENP in clone 18 (wild type cells). The *upper panel* shows that all GFP-TrAP INCENP mutants were expressed at comparable levels except for INCENP with the MyoM SAH, which was expressed at lower levels. In addition, 3D3 antibody recognizes a band at 80 kDa, which is the U35610 PTB-associated splicing factor, which shares some limited peptide sequence with the INCENP coil domain. α-Tubulin (*Tub*) serves as loading control. *C*, growth curves of cells expressing INCENP domain swap mutants in the absence of endogenous INCENP. All mutant INCENP cells show no defect in cell proliferation. GFP-TrAP-INCENP^MyoM SAH^-expressing cells and GFP-TrAP-INCENP^Double SAH^-expressing cells show a higher percentage of cell death. The average of four independent experiments is shown.

To analyze the function of the INCENP SAH domain in living cells, we used chicken DT40 conditional INCENP knock-out cells (27) to generate DT40 cell lines stably expressing a variety of INCENP domain swap mutants. The exogenous INCENP constructs were visualized by the addition of a GFP-TrAP tag at the N terminus of the proteins (28). We generated cell lines carrying the full-length wild type INCENP class I cDNA (GFP-TrAP-INCENP^WT SAH^) as well as mutants where the C-terminal half of the SAH had been deleted (INCENP^N-half SAH^), there was a duplication of the entire SAH (INCENP^Double SAH^), and a corresponding length fragment derived from the *Dictyostelium* MyoM SAH was included (INCENP^MyoM SAH^) (Fig. 2*A*). (To preserve the overall length of the coil, this was actually a duplication of the MyoM SAH.) We used the double length SAH to extend the length of the dog leash tethering Aurora B and the MyoM SAH to provide a sequence with similar physical properties but unlikely to contain protein recognition motifs in common with INCENP. We note that to accommodate reported modifications within the SAH domain (42, 43), INCENP^N-half SAH^ contained an extra19 amino acids (INCENP^598–616^) that were absent from the SAH-INCENP^503–597^ recombinant protein used for microtubule binding studies.

Based on our previous work with the INCENP conditional knock-out cells, endogenous INCENP protein becomes undetectable 26–28 h after addition of doxycycline to the medium (27). This allowed us to analyze the localization and function of INCENP mutants in the absence of endogenous INCENP. Protein levels of the GFP-TrAP-INCENP^WT SAH^ and GFP-TrAP-INCENP^N-half SAH^ domain swap mutants were similar to those of endogenous INCENP as detected by Western blotting (Fig. 2*B*). The expression level of GFP-TrAP-INCENP^Double SAH^ in the stable cell line was lower than that of endogenous INCENP, and the expression level in the GFP-TrAP-INCENP^MyoM SAH^ stable cell line was even lower (Fig. 2*B*), suggesting that those two constructs may be toxic, not fully functional, or unstable.

##### INCENP with an Altered SAH Domain Can Sustain Life of DT40 Cells

Surprisingly, all of the INCENP SAH mutant proteins tested could sustain cell proliferation in the absence of endogenous INCENP protein (Fig. 2*C*). However, cells expressing only GFP-TrAP-INCENP^Double SAH^ or GFP-TrAP-INCENP^MyoM SAH^ exhibited a higher frequency of cell death and proliferated more slowly than wild type cells. Interestingly, GFP-TrAP-INCENP^N-half SAH^ cells proliferated comparably with wild type cells, suggesting that the C-terminal half of the SAH is dispensable for INCENP function.

##### The INCENP SAH Domain Controls CPC Localization

**FIGURE 3.**
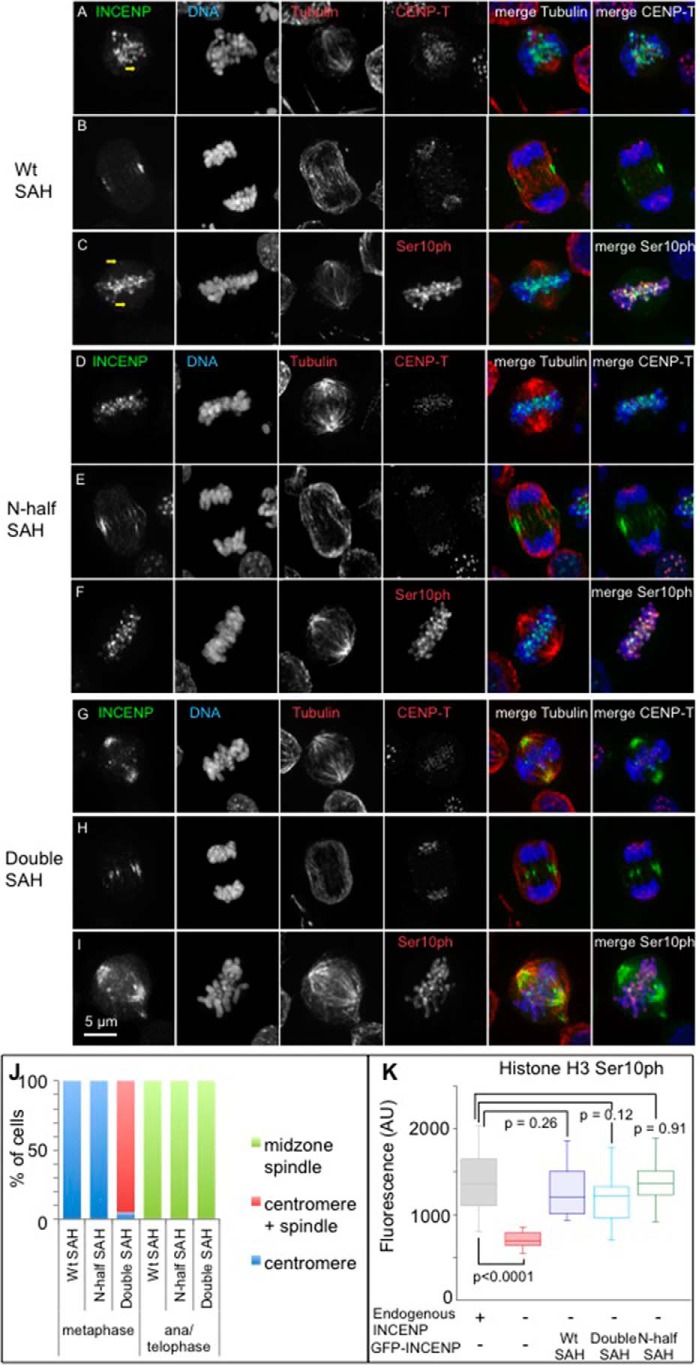
**Localization of various INCENP SAH mutants.** Micrographs of cells from stable cell lines expressing GFP-TrAP-INCENP^WT SAH^ (*A–C*), GFP-TrAP-INCENP^N-half SAH^ (*D–F*), and GFP-TrAP-INCENP^Double SAH^ (*G–I*) are shown. INCENP is shown in *green*, DNA is shown in *blue*, and tubulin or CENP-T is shown in *red. Yellow arrows* point to the faint GFP signal of GFP-TrAP-INCENP^WT SAH^ around centrosomes. *Scale bar*, 5 μm. *J*, quantification of localization of various INCENP mutants. Twenty cells were counted for each sample. The average of two independent experiments is shown. *K*, quantification of histone H3 Ser^10^ phosphorylation status of various INCENP mutants in 12–20 cells each at prometaphase. *p* values were calculated based on Student's *t* test with unpaired variants. *AU*, absorbance units.

Domain swaps of the INCENP SAH domain had complex effects on CPC localization during mitosis. GFP-TrAP-INCENP^WT SAH^ localized as expected, concentrating at the inner centromere during early mitosis (prometaphase and metaphase) and transferring to the central spindle in anaphase before finally concentrating at the midbody during cytokinesis (Figs. 3, *A–C* and *J*, and 6, *A* and *D*). Interestingly, careful examination of cells expressing GFP-TrAP-INCENP^WT SAH^ revealed that this protein also associates with centrosomes and the mitotic spindle to a minor extent at least in some metaphase cells (Fig. 3, *A* and *C*).

GFP-TrAP-INCENP^N-half SAH^ behaved exactly like the wild type. This supports the suggestion that the C-terminal half of the SAH is indeed dispensable for INCENP localization (Figs. 3, *D–F* and *J*, and 6, *B* and *E*).

GFP-TrAP-INCENP^Double SAH^ localized to centromeresduring prometaphase and metaphase, but in addition, it often decorated the mitotic spindle, concentrating strongly at the centrosomes. Central spindle and midbody localization of GFP-TrAP-INCENP^Double SAH^ at the later stages of mitosis remained similar to GFP-TrAP-INCENP^WT SAH^ (Figs. 3, *G–I* and *J*, and 6, *C* and *F*). These results suggested that having two microtubule binding sites within the SAH domain may enhance the affinity of INCENP for microtubules. Importantly, chromosome alignment at the metaphase plate appeared normal in GFP-TrAP-INCENP^Double SAH^ cells (Fig. 3, *G* and *I*).

**FIGURE 4.**
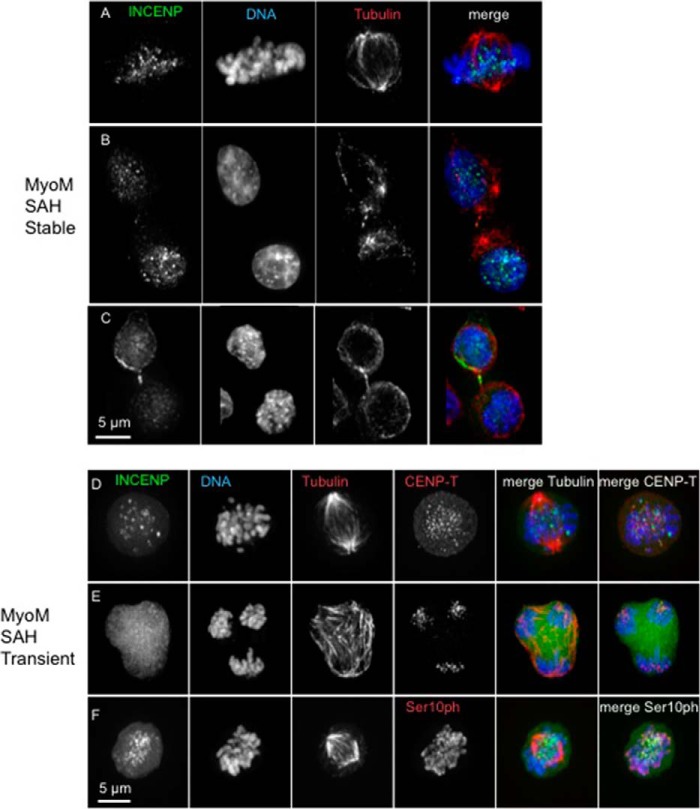
**Localization of GFP-TrAP-INCENP^MyoM SAH^ mutants.** Cells expressing GFP-TrAP-INCENP^MyoM SAH^ (*A–C*) and INCENP knock-out cells transiently transfected with GFP-TrAP-INCENP^MyoM SAH^ (*D–F*) were fixed and immunostained for 27 h following addition of doxycycline in the medium. INCENP is shown in *green*, DNA is shown in *blue*, and tubulin or CENP-T is shown in *red. Scale bar*, 5 μm.

The expression level of GFP-TrAP-INCENP^MyoM SAH^ in stable cell lines fell to extremely low levels while we were expanding the culture and was often below our detection limit. Where it could be seen, GFP-TrAP-INCENP^MyoM SAH^ was found at the inner centromere or on chromosomes during prometaphase/metaphase and occasionally at the midbody during cytokinesis in those cells (Fig. 4, *A–C*). To confirm its cell cycle localization, we transiently transfected cells with a construct expressing GFP-TrAP-INCENP^MyoM SAH^. In those cells, GFP-TrAP-INCENP^MyoM SAH^ was again occasionally seen at the inner centromere or on chromosomes, but it usually appeared diffuse throughout the entire mitotic cell (Fig. 4, *D–F*). Moreover, high levels of GFP-TrAP-INCENP^MyoM SAH^ turned out to have a disruptive effect on cell division, giving rise to tripolar spindles in most of the transfected cells 27 h after transfection in the presence of doxycycline (Fig. 4*E*). Thus, we focused our further studies on WT SAH, the N-terminal half of SAH, and double SAH mutants. The above results strongly suggest that a combination of microtubule binding activity plus flexibility is important for INCENP localization and function throughout mitosis.

##### Mutations in the INCENP SAH Domain Do Not Abolish Aurora B Kinase Activity but Regulate CPC Localization

INCENP is the scaffolding subunit of the chromosome passenger complex of which Aurora B kinase is the catalytic subunit (12, 44). To determine whether the catalytic activity of the CPC is compromised by changes within the SAH domain, we stained cells expressing various SAH domain swaps with antibody against H3Ser^10^ph. H3Ser^10^ph levels appeared similar in all mutant cells (Figs. 3, *C*, *F*, *I*, and *K*, and 4*F*). Thus, Aurora B kinase activity was not impaired by these modifications of the INCENP SAH domain.

**FIGURE 5.**
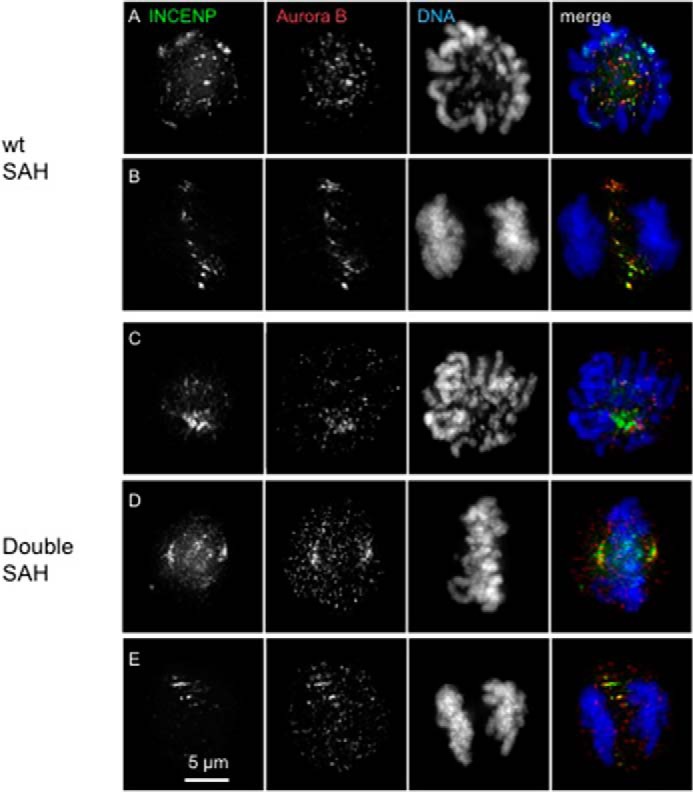
**Aurora B kinase co-localizes with INCENP SAH mutants.** Micrographs of cells from stable cell lines expressing GFP-TrAP-INCENP^WT SAH^ (*A* and *B*) and GFP-TrAP-INCENP^Double SAH^ (*C–E*) are shown. Cells were fixed and immunostained 27 h following addition of doxycycline in the medium. INCENP is shown in *green*, DNA is shown in *blue*, and Aurora B is shown in *red. Scale bar*, 5 μm.

We next investigated whether the INCENP SAH mutants affected the localization of Aurora B kinase. In cells expressing GFP-TrAP-INCENP^WT SAH^, Aurora B co-localized with INCENP, concentrating at the centromeres during early stages of mitosis and then transferring to the central spindle during mitotic exit (Fig. 5, *A* and *B*). In cells expressing GFP-TrAP-INCENP^Double SAH^, Aurora B again co-localized with INCENP, decorating the mitotic spindle close to centrosomes and then transferring to the central spindle during mitotic exit (Fig. 5, *C–E*). The above results indicated that the INCENP SAH domain influences the localization of not only INCENP but also the rest of the CPC complex. This prompted us to use the INCENP SAH mutants to test the dog leash model of CPC regulation.

##### INCENP SAH Length Influences Aurora B Substrate Phosphorylation

According to the flexible dog leash model (a term first proposed by Santaguida and Musacchio (9)), Aurora B kinase bound to the INCENP C terminus can move freely even though the centromere-targeting module of the CPC including the N terminus of INCENP is tethered to static nucleosomes during early mitosis (Fig. 7). If this model is correct, a short SAH domain (N-half) should favor phosphorylation of substrates proximal to the inner centromere but should disfavor phosphorylation of substrates further away, such as those in the outer kinetochore.

**FIGURE 6.**
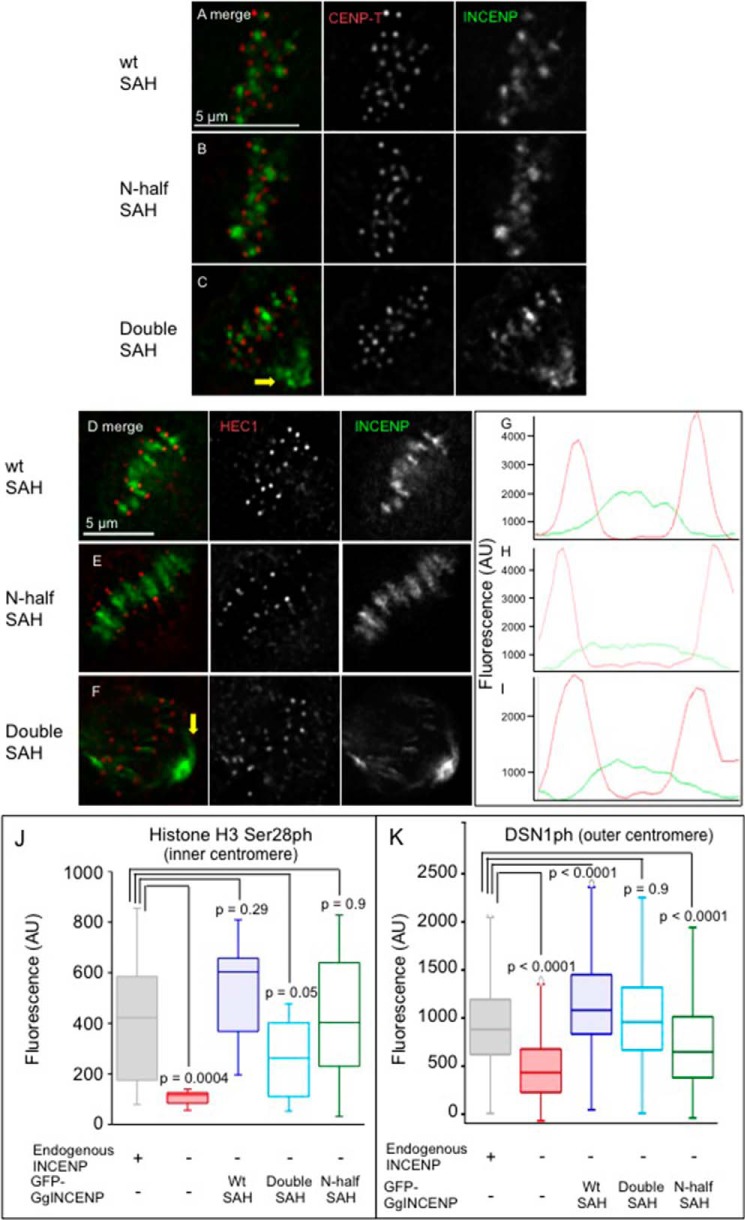
**Phosphorylation status of inner/outer kinetochore proteins affected by INCENP SAH mutants.** Micrographs of chicken DT40 stable cell lines expressing GFP-TrAP-INCENP^WT SAH^ (*A*), GFP-TrAP-INCENP^N-half SAH^ (B), and GFP-TrAP-INCENP^Double SAH^ (*C*) are shown. Cells were fixed and immunostained 27 h following addition of doxycycline in the medium. Micrographs show HeLa cells transiently transfected with various GFP-TrAP-GgINCENP mutants and siRNA against endogenous INCENP. *D* and *G*, GFP-TrAP-INCENP^WT SAH^; *E* and *H*, GFP-TrAP-INCENP^N-half SAH^; *F* and *I*, GFP-TrAP-INCENP^Double SAH^. *G–I*, representative line profiles across paired kinetochores. INCENP is shown in *green*, CENP-T or HEC1 is shown in *red. Scale bar*, 5 μm. *Yellow arrows* point to the pool of GFP-TrAP-INCENP^Double SAH^ on mitotic spindle close to centrosomes. The phosphorylation status of histone H3 Ser^28^ (inner centromere) (*J*) or DSN1 (outer kinetochore) (*K*) was quantified in 18–20 cells each at prometaphase. *p* values were calculated based on Student's *t* test with unpaired variants. *AU*, absorbance units.

Despite extensive efforts, we failed to obtain any phosphospecific antibodies recognizing Aurora B substrates in the outer kinetochore of chicken DT40 cells. However, we noted that GFP-tagged INCENP SAH mutants all localized normally to inner centromeres in HeLa cells (Fig. 6, *D–I*) as they did in DT40 cells (Fig. 6, *A–C*). We carried on image analysis and quantification of phosphorylation using HeLa cells transiently transfected with GFP-TrAP-GgINCENPs and treated with siRNA oligonucleotides to deplete endogenous HsINCENP. H3Ser^28^ph and DSN1ph (MIS12 complex) were chosen as representative substrates of Aurora B kinase in the inner centromere and outer kinetochore, respectively.

As expected, phosphorylation of both H3Ser^28^ and DSN1 was significantly reduced after depletion of HsINCENP but could be rescued by expression of GFP-TrAP-GgINCENP^WT^ (Fig. 6, *J* and *K*). Expression of INCENP^Double SAH^ was substantially less effective at phosphorylating H3Ser^28^ (the inner centromere marker). In contrast, expression of INCENP^N-half SAH^ was less effective at rescuing phosphorylation of DSN1 (the outer kinetochore marker). These results show clearly that the length of the INCENP SAH could influence the phosphorylation status of inner centromere and outer kinetochore substrates of Aurora B kinase.

## Discussion

Since the original analysis of the INCENP amino acid sequence, the central portion of INCENP class I (Gg residues 503–717) has been assumed to form a coiled coil structure (37, 45). This region has been found to be required for interactions with microtubules and to play a role in the spindle assembly checkpoint functions of the CPC (24, 37, 46). However, the exact role of the INCENP putative coiled coil in CPC regulation and function remained largely unclear (24, 37, 46, 47). Here we demonstrate that amino acids 503–717 of GgINCENP behave *in vitro* as an SAH domain whose N-terminal region directly binds to microtubules *in vitro*.

### 

#### 

##### INCENP Contains a Monomeric SAH Domain

Single α-helices are generally thought to be inherently unstable in aqueous solution (48). However, the INCENP coil region forms a highly helical, monomeric SAH domain that is relatively stable over a broad range of temperatures, salt concentrations, and pH values. Similar results have been obtained for the SAH domain of myosin 10 and other SAH domains (20, 49, 50). The 97-amino acid MyoM SAH domain is thought to behave as a constant force spring as described for the SAH domain of myosin 10 (20). The myosin10 SAH domain unfolds non-cooperatively at very low forces (<30 pN) from a fully folded length of 14.5 nm to a completely extended coil structure with a length of 37 nm with very little additional increase in force. Moreover, it can refold when the force exerted on it is reduced. INCENP has an even longer SAH domain (213 amino acids). As a result, the INCENP SAH can likely alter its length from a resting length of ∼32 nm long (based on a rise per residue of 0.15 nm) to as long as ∼80 nm (∼0.36 nm per residue when unfolded), thereby acting as a highly flexible linker between its flanking domains. This elastic feature of a monomeric INCENP SAH domain has not previously been incorporated into models explaining the dynamic localization and function of the CPC.

##### The INCENP SAH Domain Binds Microtubules

INCENP has at least two direct microtubule-binding sites (37, 39, 40, 51, 52). One, located near the N terminus of the protein, is negatively regulated by CDK1 phosphorylation and functions *in vivo* only after anaphase onset (53, 54). We have found that the other, located in the N-terminal half of the SAH domain, GgINCENP^503–597^, appears to function in early mitosis. Consistent with its possessing an extra microtubule-binding site, the GFP-TrAP-INCENP^Double SAH^ domain swap mutant often associates with the entire mitotic spindle and accumulates around centrosomes during early mitosis, taking Aurora B along with it. Aurora B kinase has been reported previously to localize and be active on mitotic spindles in human telomerase reverse transcriptase RPE1 cells and on mitotic spindles of *Xenopus* egg extracts (24).

Interestingly, localization of GFP-TrAP-INCENP^Double SAH^ at centrosomes ceased at the metaphase/anaphase transition, and the protein concentrated on the central spindle similarly to wild type INCENP. This suggests either that the INCENP SAH domain loses its microtubule binding activity at anaphase onset or possibly that MKLP2-mediated INCENP translocation to the central spindle (54) becomes dominant.

##### Roles of the INCENP SAH Dog Leash

Aurora B activation is thought to be promoted by INCENP clustering in the inner centromere and on spindle microtubules (12, 22, 24, 40, 55). However, this mechanism suggests a conundrum that has apparently not been considered previously. Formation of an INCENP coiled coil would presumably involve INCENP dimerization and therefore the dimerization of CPC complexes. If that is true, each complex would contain two Aurora B molecules, which would presumably be free to trans-phosphorylate the partner INCENP and one another, thereby autoactivating the CPC with no need for microtubule or chromosome association (12, 23, 24, 40, 55). Thus, coiled coil formation would have to be carefully regulated. If instead the INCENP coil is an SAH this concern is eliminated, and existing models of CPC activation are readily explained.

**FIGURE 7.**
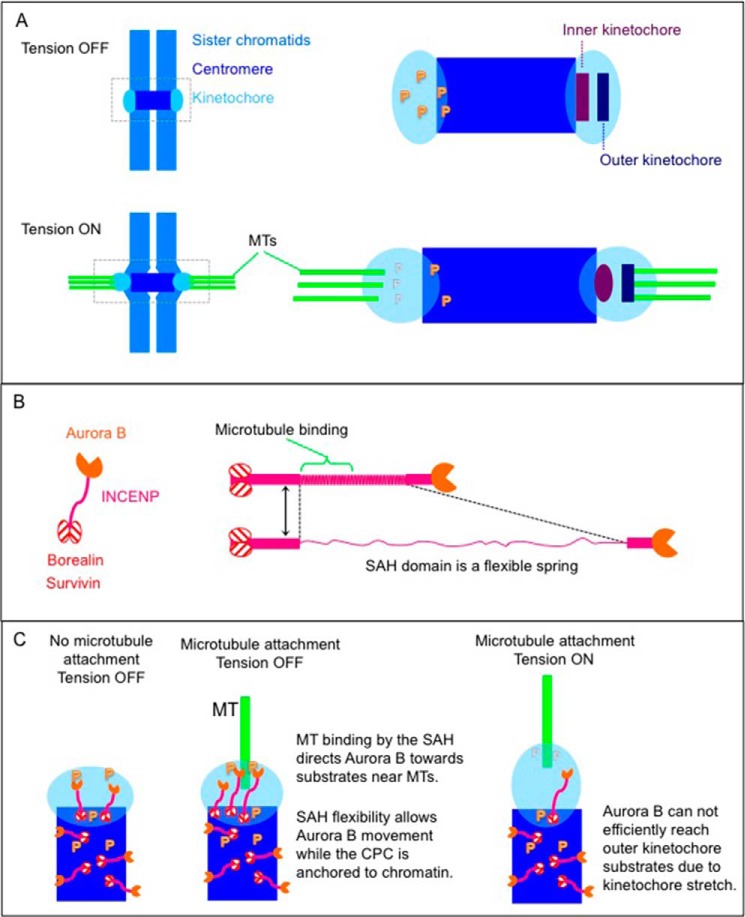
**Model for INCENP action.** *A*, outer and inner kinetochore proteins are heavily phosphorylated when tension from mitotic spindle is off. When bipolar attachment of microtubules to kinetochore is established, the centromere is stretched, and outer kinetochore proteins are less phosphorylated. *B*, INCENP, Borealin, Survivin, and Aurora B form the CPC. The INCENP SAH domain consists of 213 amino acids. The N-terminal half of the INCENP SAH domain binds to microtubules. *C*, the CPC phosphorylates inner/outer kinetochore proteins. The N terminus of INCENP, associated with Borealin and Survivin, tethers the CPC to the inner centromere. Microtubule binding activity within the INCENP SAH domain directs Aurora B toward substrates near microtubules (*MT*). Flexibility within the SAH domain allows Aurora B to follow substrates on dynamic microtubules while protecting the integrity of the CPC complex. Aurora B cannot reach its outer kinetochore substrates when tension is fully on.

In addition to solving the activation problem, we suggest that the flexible dog leash (a term first proposed by Santaguida and Musacchio (9)) is also an efficient mechanism to allow Aurora B molecules to move freely over regions of tens of nanometers even though the targeting module of the CPC is tethered to static nucleosomes during early mitosis (Fig. 7). This might enable the kinases to find one another and perform the trans-phosphorylation that produces full kinase activation.

Importantly, the forces required for extension of an SAH are in the range provided by interactions between microtubules and the kinetochore. The myosin 10 SAH domain can extend by up to ∼2.5-fold when forces of less than 30 pN are exerted on it (20). Single microtubule protofilaments can generate up to 5 pN during depolymerization, and it has been proposed that a single microtubule (composed of 13 protofilaments) can produce a force of 65 pN (56). One yeast kinetochore complex can associate with one microtubule persistently supporting loads up to 11 pN (57, 58). In vertebrates, kinetochores associate with ∼4–20 microtubules. Thus, the aggregate forces exerted within and around kinetochores are well above the ∼30 pN required to extend an SAH peptide. Interestingly, coiled coils can also exhibit the behavior of constant force springs, and the myosin coil can be extended by about 2–2.5 times its original length at forces of 20–25 pN (59).

Because microtubules bind to the N-terminal half of the SAH, extension of this portion of the SAH domain (we estimate that an ∼20-nm extension is possible given the parameters assumed above and a length of 92 amino acids) could bridge the gap between the three-helix CPC-targeting module associated with chromatin and microtubules in the outer kinetochore (Fig. 7). The flexible C-terminal half of the SAH domain might then allow Aurora B to remain associated with kinetochore substrates, such as the Ndc80 and Ska complexes, which presumably significantly alter their conformations as microtubules grow and shrink during chromosome oscillations. Overall, the SAH domain could act as a shock absorber, allowing CPC to remain associated with dynamic substrates close to microtubules while at the same time being docked to static chromatin.

The distance between CENP-A and the C-terminal region of the Ndc80 complex can extend from 65 to 100 nm under tension in *Drosophila melanogaster* S2 cells in a process referred to as intrakinetochore stretch (60). HeLa chromosomes were also shown to undergo a similar stretch (61). In chicken DT40 cells, the width of the inner kinetochore extends by 35 nm, and the width of the outer kinetochore extends by 28 nm under tension (62). In human cells, intrakinetochore stretch is typically around 20 nm but can extend as far as 60 nm (63). Thus, these extensions tend to be slightly greater than the ∼20-nm extension allowed by stretching the N-terminal half of the INCENP SAH. This might explain how Aurora B kinase can reach substrates in the outer kinetochore when the kinetochore is under no or low tension but is not able to reach those substrates when the kinetochore is maximally stretched, thereby stabilizing kinetochore-microtubule interactions. Our study provides experimental support for this model by showing clearly that the length of the INCENP SAH can influence the phosphorylation status of inner centromere and outer kinetochore substrates of Aurora B kinase.

It is now clear that the SAH domain of INCENP is one of several factors regulating the dynamic CPC localization and functions during mitosis. A challenge for the future will be to combine established structural techniques, such as crystallography and electron cryomicroscopy, with emerging methods, such as cross-linking with mass spectrometry, to fully characterize the structural basis of CPC regulation of kinetochore function.

## Author Contributions

K. S. and M. Pi. designed and performed the study and wrote the paper. M. W. designed and performed all of the *in vitro* analysis of INCENP SAH domain except the microtubule binding assay. H. O. established cells lines and performed the study. G. V. helped with subcloning and the initial characterization of the cell lines. P. K., M. Pe., and W. C. E. wrote the paper. All authors reviewed the results and approved the final version of the manuscript.

## Footnotes

6 The abbreviations used are:

INCENP: inner centromere protein
SAH: single α-helix
CPC: chromosome passenger complex
Gg: *Gallus gallus*
Hs: *Homo sapiens*
TrAP: triple affinity purification
MyoM: myosin M
*TRITC*: tetramethylrhodamine isothiocyanate
CENP: centromere protein
pN: piconewtons
H3: histone H3
ph: phosphorylation.
